# Fibrin Sealant: The Only Approved Hemostat, Sealant, and Adhesive—a Laboratory and Clinical Perspective

**DOI:** 10.1155/2014/203943

**Published:** 2014-03-04

**Authors:** William D. Spotnitz

**Affiliations:** Surgical Therapeutic Advancement Center (STAC), Department of Surgery, University of Virginia Health System, P.O. Box 801370, Charlottesville, VA 22908-1370, USA

## Abstract

*Background.* Fibrin sealant became the first modern era material approved as a hemostat in the United States in 1998. It is the only agent presently approved as a hemostat, sealant, and adhesive by the Food and Drug Administration (FDA). The product is now supplied as patches in addition to the original liquid formulations. Both laboratory and clinical uses of fibrin sealant continue to grow. The new literature on this material also continues to proliferate rapidly (approximately 200 papers/year). *Methods.* An overview of current fibrin sealant products and their approved uses and a comprehensive PubMed based review of the recent literature (February 2012, through March 2013) on the laboratory and clinical use of fibrin sealant are provided. Product information is organized into sections based on a classification system for commercially available materials. Publications are presented in sections based on both laboratory research and clinical topics are listed in order of decreasing frequency. *Results.* Fibrin sealant remains useful hemostat, sealant, and adhesive. New formulations and applications continue to be developed. *Conclusions.* This agent remains clinically important with the recent introduction of new commercially available products. Fibrin sealant has multiple new uses that should result in further improvements in patient care.

## 1. Introduction

Fibrin sealant is a two-component material consisting of fibrinogen and thrombin. In the presence of small amounts of calcium and factor XIII, the thrombin converts fibrinogen into insoluble fibrin, the final stable form of the agent. Fibrin sealant now has over a century of development and use. Bergel used fibrin first as a hemostat in 1909 [1], Young and Medawar used it as an adhesive in 1940 [2], Matras used concentrated fibrinogen for nerve attachment in 1972 [3], and the Food and Drug Administration (FDA) approved liquid fibrin sealant in 1998 [4, 5] as well as a fibrin sealant patch in 2010 [6, 7].

**Figure 1:**
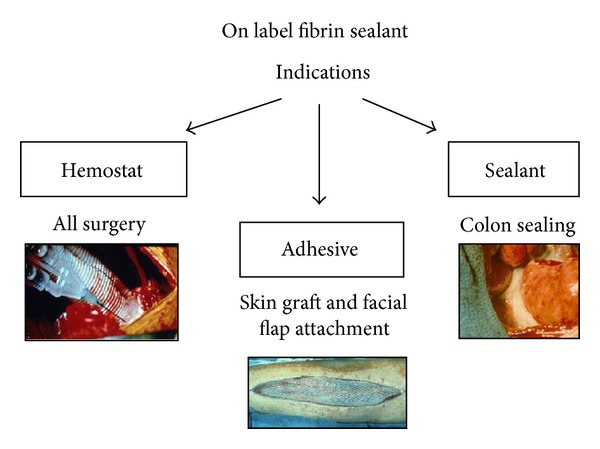
The current FDA approved indications for the use of fibrin sealant [8], adapted and reprinted with kind permission from Springer Science + Business Media in [8, Page 633, Figure 1].

Although straightforward, a few clear definitions are important in this area. A hemostat causes blood to clot and usually is not effective unless blood is present in the operative field. A sealant creates a sealing barrier that prevents the leakage of gas or liquid from a structure. It polymerizes on its own and is often most effective in a dry field. An adhesive is capable of gluing structures together. It is also self-polymerizing and usually most effective in a dry field. Both sealants and adhesives when applied to potentially leaking blood vessels may have a hemostatic effect by blocking holes in the vessel and preventing bleeding, but they do not necessarily cause blood to clot. Fibrin sealant is the only commercially available FDA approved material for clinical use in all the three of these groupings: hemostats, sealants, and adhesives (Figure 1).

For a period of 25 years, it was my own good fortune to have the opportunity to perform both laboratory [9–24] and clinical research on fibrin sealant [25–41]. These efforts included work in a variety of surgical disciplines including breast [9, 13, 14, 16, 17, 32, 36], cardiovascular [11, 26, 27, 30, 33], gastrointestinal [10], head and neck [22, 35], hepatic [12, 20], neuro- [29], orthopedic [21, 37, 40, 41], pediatric [12], noncardiac thoracic [25, 30, 31, 33, 34], and vascular surgery [15, 18, 19, 23, 24, 38, 39]. This body of work emphasized the evolving versatility of fibrin sealant because it is capable of causing blood to clot, creating a sealing barrier, and gluing tissues together. Also, these research efforts clarified that fibrin sealant often requires some hands on experience with the agent to maximize its effectiveness [40].

A system of classification for the FDA approved hemostats, sealants, and adhesives has been developed and helps in understanding not only the available commercial products, but also the amazingly diverse abilities of fibrin sealant [42–45]. This system as noted in Table 1 is based on three major groupings: the hemostats, sealants, and adhesives. Each of these groups is first broken down into categories and then into classes. As emphasized (bold font), it is immediately evident that fibrin sealant is the only commercially available material present in all three groups and that it is the only material available in liquid as well as patch forms (italics). Thus, fibrin sealant is the only material that has been extensively studied and evaluated in all three applications (as a hemostat, sealant, and adhesive) and in multiple formulations (liquid and patch). The commercial fibrin sealants currently available in the USA are illustrated (Table 2). These commercial products are described in the following sections based on their safety, efficacy, usability, and cost [42–45].

## 2. FDA Approved Hemostats

The fibrin sealant category of hemostats [45] is divided into four classes as noted in Table 1: human pooled plasma fibrinogen and thrombin, individually obtained units of human plasma (possibly platelet-enriched) mixed with bovine thrombin and collagen, and dry human pooled plasma fibrinogen and thrombin fixed on either an equine collagen or oxidized regenerated cellulose patch.

### 2.1. Human Pooled Plasma Fibrinogen and Thrombin

The safety of these liquid agents is most dependent on the fact that multiple donor plasma pools are used for their preparation (Tisseel, Baxter, Westlake Village, CA; Evicel, Ethicon/J&J, Somerville, NJ) [46, 47]. Both contain highly concentrated forms of fibrinogen and thrombin, respectively (Tisseel, 85 mg/mL, 500 IU/mL : Evicel, 70 mg/mL, 1,000 IU/mL) derived from large pools of human plasma. Thus, these forms of human pooled plasma fibrin sealant may be associated with viral disease (Parvovirus B19, hepatitis, HIV) or prion disease (CJD {Creutzfeldt Jakob}) transmission [46, 47]. Reports of Parvovirus B19 transmission have appeared from the Japanese literature [48, 49] because this virus is particularly difficult to remove from plasma. One paper suggested that viral transmission could occur in up to 20% of patients receiving pooled plasma fibrin sealant [49]. However, it has been emphasized by others that no reports of hepatitis or HIV transmission with fibrin sealant have appeared in over 20 years of the world literature because of extensive viral prevention methods including viral screening (serology and polymerase chain reaction {PCR} testing) and viral reduction methods including filtration, heat treatment (dry or vapor heating, pasteurization), solvent/detergent cleansing, precipitation, pH treatment, and chromatography [50]. Additional safety concerns include the fact that any product containing thrombin such as fibrin sealant should never be injected intravascularly because such administration may be associated with thrombosis, hypotension, and death. Similarly, fibrin sealant should not be allowed to enter cell saver or cardiopulmonary bypass circuits because of the risk of thrombosis [45]. Further safety concerns [46, 47] include the danger of applying too much fibrin sealant which may contribute to infection and reduced healing as well as air emboli associated with use of gas driven sprayers supplied by product manufacturers. Increased risk of infection may result when a fibrin protein growth medium for bacteria is created. Increased risk of tissue necrosis may occur if diffusion of nutrients and fibroblast ingrowth is blocked by an excessively thick layer of fibrin sealant. Air emboli may occur when air from the gas powered spray applicator is driven into central veins particularly when the fibrin sealant is applied against manufacturer recommendations at higher pressure (>20–25 psi) or shorter separation distance (<10–15 cm). One agent (Tisseel, Baxter, Westlake Village, CA) may be associated with skin rash, allergy, or anaphylaxis based on the inclusion of aprotinin (3,000 KIU/mL) as an antifibrinolytic in the formulation [46] and both pooled plasma liquid fibrin sealants (Tisseel, Baxter, Westlake Village, CA; Evicel, Ethicon/J&J, Somerville, NJ) may be associated with allergic reaction or anaphylaxis due to exposure to human proteins. Biodegradation occurs over a period of 10–14 days [46].

The efficacy of pooled plasma liquid fibrin sealant as a hemostat has been demonstrated in at least 14 multicenter, prospective, randomized trials [37, 39–41, 51–61], and all of these commercial products [46, 47] now have broad label approvals for hemostasis. These clinical trials included clinically relevant and statistically significant findings in multiple surgical specialties: burn [54], cardiac [51, 57], general [60], pediatric (cannulation for extracorporeal oxygenation {ECHMO}) [52], hepatic [56], orthopedic [37, 40, 41, 53], and vascular surgery [39, 55, 58, 59, 61].

Since its approval in 1998, liquid pooled plasma fibrin sealant has been relatively difficult to be reconstituted and used in the operating room [45]. The need for thawing [46, 47] and mixing [46] reduced the willingness of operating room staff to prepare these agents particularly in emergent situations. Recent advances have allowed one product (Tisseel, Baxter, Westlake Village, CA) [46] to come fully mixed and assembled in dual syringe applicators with only a brief period of thawing (as little as 5 minutes) required. The other product (Evicel, Ethicon/J&J, Somerville, NJ) is provided, fully mixed, and is storable after thawing for up to one month in the refrigerator ready for prompt use [47]. These liquid forms of fibrin sealant are provided with drip applicators for local placement and gas driven spray applicators for covering larger surface areas [45]. Laparoscopic attachments are also available [45]. Use of gas driven sprayers particularly in the laparoscopic setting should be accompanied by significant caution. One group of investigators performed extensive evaluations in a pig model and suggested using only brief periods of spray application (<10 seconds) while allowing gas to escape from the insufflated abdomen via the trocar (recommended initial insufflation pressure 10 mmHg, gas pressure 2.5 bars, application distance 5 cm, and trocar valve open) [62] to avoid excessive elevations of intra-abdominal pressure. Another group of investigators has warned about the risk of subcutaneous emphysema associated with laparoscopic gas driven fibrin sealant application [63]. These authors suggested preferentially using CO_2_ for a gas source as it dissolves relatively rapidly in human blood and soft tissues as compared to air or nitrogen [63]. My own most effective method of applying these liquid agents has been in combination with absorbable gelatin sponge to create a patch [45, 51, 64] that after application with several minutes of pressure should be left in place.

Liquid pooled plasma fibrin sealants are in the more expensive range of hemostats presently available. These commercial products cost about $50/mL of final mixed fibrin sealant [45].

### 2.2. Individually Obtained Units of Human Plasma (or Platelet-Enriched Plasma) Mixed with Bovine Thrombin and Collagen

This class of fibrin sealants contains one commercial product (Vitagel, Stryker, Malvern, PA). This form of fibrin sealant comes as a kit containing bovine collagen (20 mg/mL) and thrombin (300 IU/mL in 40 mM CaCl_2_ buffer) as well as the materials required to obtain plasma from patient's blood [65]. The manufacturer (Stryker, Malvern, PA) now recommends that the plasma used be the platelet-enriched fraction to take advantage of the role of platelets in hemostasis and healing [66]. The safety of this material is most strongly influenced by the fact that bovine thrombin is used instead of human thrombin to convert plasma fibrinogen to fibrin. Although not associated with human viral transmission, bovine thrombin may be associated with an immune mediated coagulopathy. Bovine thrombin (Thrombin-JMI, King, Pfizer, UPM Pharmaceuticals, Bristol, TN) carries an FDA black box warning that its use may lead to occasional laboratory clotting abnormalities and coagulopathy as well as death in rare instances [67]. These risks are related to the patient forming antibodies against the bovine factor II (thrombin) provided in the kit and the small amounts of bovine factor V present in bovine thrombin [67] as a contaminant. The risk of coagulopathy becomes more significant on repeated exposure. The antibovine antibodies formed by humans in response to the bovine clotting factor antigens are in some cases capable of cross-reacting and neutralizing human factor II and V thereby blocking the common pathway of the clotting cascade. In addition, the bovine microfibrillar collagen present in this particular fibrin sealant formulation may be associated with an increase in bovine serum antibodies as well [68]. The use of this form of fibrin sealant may also be associated with swelling due to the bovine collagen that may be particularly important in closed bony spaces or in contact with the central nervous system (CNS) [68]. However, recent data from the manufacturer (Stryker, Malvern, PA) suggests that this product does not swell and can be left safely at the operative site [69]. Biodegradation of this fibrin sealant occurs over 30 days [65].

The effectiveness of this product has been demonstrated in a multicenter, prospective, randomized trial [65, 70] including cardiac, hepatic [71], general surgical, and orthopedic patients (cancellous bone bleeding) [72]. Large, clinically, and statistically significant improvements in the time to hemostasis were found in all specialty groups. This agent has a broad regulatory approval for surgical hemostasis.

The blood donation and centrifugation required with this fibrin sealant formulation in order to obtain the individual units of donor plasma required may be labor intensive. Some operating rooms have technicians or perfusionists who are experts in the procedures needed to obtain donor plasma. In such a setting, the system does allow for the production of fibrin sealant from autologous units of platelet-enriched plasma in combination with the bovine collagen and thrombin provided in the manufacturer's kit [66]. Applicators include a malleable drip tip, an extended laparoscopic device, and a spray system [66].

The cost of this product kit is reported to be in the range of $600 to produce 10 mL of final fibrin sealant product [73].

### 2.3. Dry Human Pooled Plasma Fibrinogen and Thrombin Fixed on an Equine Collagen or Oxidized Regenerated Cellulose Patch

These two separate classes of fibrin sealant hemostats will be discussed together because they differ predominantly only in the material chosen to create the patch. Their safety profiles (Tachosil, Baxter, Westlake Village, CA; Evarrest, Ethicon/J&J, Somerville, NJ) are similar to those of the pooled plasma liquid fibrin sealants discussed previously [46, 47] earlier in this review and are related to viral and prions disease transmission [74, 75]. Thus, the risks associated with using pooled plasma will not be discussed again in detail in this section. Neither of the available patches should be packed into tight spaces to avoid compression injuries, placed in infected or contaminated spaces to avoid further increases in infection, nor placed intravascularly to avoid life threatening thromboembolic events [74, 75]. Both patches may cause allergic or anaphylactic reactions to the human serum proteins used in the preparations.

The equine collagen as patch (Tachosil, Baxter, Westlake Village, CA) may cause an allergic reaction in patients' sensitive to horse proteins [74] and the oxidized regenerated cellulose patch (Evarrest, Ethicon/J&J, Somerville, NJ) may be associated with adhesion formation [74]. Neither patch contains an antifibrinolytic agent and thus avoids some of the potential complications associated with the use of these stabilizers [42–45]. Biodegradation occurs over a period of 8 (Evarrest, Ethicon/J&J, Somerville, NJ) [75] to 13 (Tachosil, Baxter, Westlake Village, CA) [74] weeks.

The fibrin sealant equine collagen patch (Tachosil, Baxter, Westlake Village, CA) is presently FDA approved as a hemostat for use in only cardiac surgery. This patch has been evaluated in multicenter, prospective, randomized clinical trials in cardiac [76], hepatic [77, 78], and renal operations [79] with clinically and statistically significant benefit in achieving hemostasis in all four investigations.

The fibrin sealant oxidized regenerated cellulose patch (Evarrest, Ethicon/J&J, Somerville, NJ) is presently FDA approved as a hemostat only in soft tissue surgery. A multicenter, prospective, randomized trial of soft tissue application during retroperitoneal, intra-abdominal, pelvic, and noncardiac thoracic procedures has been completed with clinically and statistically significant benefit in achieving hemostasis in all four arms of the investigation [75].

A major advantage of both patches [74, 75] is that they do not require complex storage, specifically freezing or refrigeration, and are immediately ready for use after opening a simple package. No reconstitution or device loading is necessary making these forms of fibrin sealant highly attractive to operating room personnel particularly in emergent situations. The equine collagen patch (Tachosil, Baxter, Westlake Village, CA) contains riboflavin that provides a yellow color to the active side while the oxidized regenerated cellulose patch (Evarrest, Ethicon/J&J, Somerville, NJ) appears powdery on the active side (fibrinogen and thrombin embedded in a layer of polyglactin 910 {PG910} nonwoven fibers) as opposed to its nonactive side which is embossed with a wavy pattern (any adherence of the this nonactive side can be reduced by moistening with saline). The active sides (Tachosil, yellow: Evarrest, powdery nonembossed side) of the patches are placed on the bleeding site and pressure is applied to the entire patch for at least 3 minutes [74, 75]. The patches must be left in place as they become adherent to the wound. The patches are provided in variety of sizes (Tachosil, 9.5 × 4.8, 4.8 × 4.8, and 3.0 × 2.5 cm : Evarrest 10.2 × 10.2 cm) and are embedded with different concentrations of fibrinogen and thrombin, respectively (Tachosil, 5.5 mg and 2.0 U per cm^2^ : Evarrest 7.8 mg and 31.5 U per cm^2^). The manufacturers recommend specific restrictions on the number of patches to be placed that depend on the size of the patch being used.

These patches are the most costly hemostats presently approved by the FDA with prices reportedly approaching $800 per patch.

## 3. FDA Approved Sealant

The fibrin sealant category of sealants [45] consists of one class containing one FDA approved commercial product as noted in Table 1.

### 3.1. Human Pooled Plasma Fibrinogen and Thrombin

The single product in this class (Tisseel, Baxter, Westlake Village, CA) has been described previously in Section 2.1. Thus, the safety profile as well as usability and cost issues will not be repeated here as they are as described above.

With respect to efficacy as a sealant, this particular form of fibrin sealant is approved by the FDA as a sealant to prevent leakage of bowel contents at the time of colostomy closure [46]. Using this product to seal the colon was demonstrated in a single center, prospective, randomized trial to provide statistically significant reductions in complications associated with bowel anastomoses including leakage, abscess, reoperation, shock, and death [46]. No multicenter prospective randomized trials have been published in this indication [45]. When being used for intestinal sealing in my experience, fibrin sealant has only moderate strength and should be applied to as dry a surface as possible to maximize effectiveness [46].

## 4. FDA Approved Tissue Adhesive

The fibrin sealant category of adhesives [45] consists of one class containing one FDA approved commercial product as noted in Table 1.

### 4.1. Human Pooled Plasma Fibrinogen and Thrombin

The single product in this class (Artiss, Baxter, Westlake Village, CA) consists of concentrated fibrinogen (85 mg/mL), thrombin (5 IU/mL), and synthetic aprotinin (3,000 KIU/mL). It is similar in safety profile [80] to the pooled plasma fibrin sealant hemostat and sealant (Tisseel, Baxter, Westlake Village, CA) described previously [46] in Sections 2.1 and 3.1. Thus, the entire safety profile will not be repeated here because it is as described above. One key safety concern in this adhesive application is the danger of applying too thick layer prior to placement of the skin graft or flap that may prevent nutrient diffusion to the graft or healing of the flap [80].

The FDA has approved this adhesive for placement of skin grafts at burn debridement sites as a replacement for sutures or staples and for skin flap attachment at the time of rhytidectomy (face lift) procedures [80]. The thrombin concentration when thrombin is combined with fibrinogen directly affects the rate of fibrin formation [81]. Because this formulation contains a lower thrombin concentration (5 IU/mL) than other forms of fibrin sealant used either as hemostats or adhesives, it polymerizes more slowly over a period of one minute allowing time for proper graft or flap placement [80]. Two multicenter, prospective, randomized trials [80, 82, 83] support the use of this fibrin sealant for skin graft and flap attachment. In the skin graft study [82], patients served as their own controls (two different similar size and location burns) comparing graft attachment at burn sites with fibrin sealant versus skin staples. The quality of graft attachment as measured by wound closure at day 28 was judged to be noninferior for the fibrin sealant. In addition, there were statistically significant reductions in hematoma and seroma formation in the fibrin sealant group at day 1 (*P* < 0.0001) and the participating investigators noted statistically significant benefit using the fibrin sealant for graft: adherence quality, fixation method preference, satisfaction with fixation; and overall healing (all *P* < 0.0001). Patient determined outcomes also favored fibrin sealant with less anxiety and a preference for use of the sealant over staples (both *P* < 0.0001). In the second study [83] using facelift patients as their own controls (opposite sides of the face), there were statistically significant reductions using fibrin sealant in the volume of serous fluid output (7.7 ± 7.4 mL as compared to 20.0 ± 11.3 mL, *P* < 0.0001).

The usability of this fibrin sealant adhesive is strongly affected by the additional time (60 seconds) available for manipulation of grafts or flaps in order to assure proper placement. The additional time to complete polymerization offers the advantage of allowing time to properly position grafts or flaps. It should be remembered, however, that this additional time to complete polymerization may allow more time for the liquid fibrin sealant to migrate to the most dependent portions of the wound before sticking particularly in wounds with large topographic differences resulting in an irregular final layer of application. After positioning of the graft or flap, gentle pressure is applied for 3 minutes to assure proper adherence. No additional manipulation of the graft or flap should then occur to prevent disruption of the fibrin sealant adhesive bonds as if this occurs the incidence of seroma formation and drainage volume will increase tremendously [45] as the fibrin sealant actually begins to function as an antiadhesive. In terms of storage, reconstitution, and applicators, this form of fibrin sealant (Artiss, Baxter, Westlake Village, CA) is similar to that reviewed for liquid fibrin sealant (Tisseel, Baxter, Westlake Village, CA) earlier in this review [46]. There are two available formulations. The room temperature lyophilized powder kits require reconstitution including mixing while the different sizes (2, 4, and 10 mL kits) of mixed frozen fibrin sealant in preloaded syringes require different thawing times which are the fastest in a sterile water bath (33°–37°C) on the operative field (5, 5, and 12 minutes, resp.). The thawed unopened frozen pouches may be kept for up to two weeks in a refrigerator [80] for prompt use.

This fibrin sealant is similar in cost to those commercial pooled plasma liquids approved as hemostats [46, 47], approximately $50/mL of final mixed product.

## 5. Non-FDA Approved Formulations of Fibrin Sealant

A variety of methods for obtaining the fibrinogen and thrombin required for fibrin sealant are available although not FDA approved for any specific indications including ammonium sulfate [84] or cold [85] precipitation. The most frequently employed alternatives to the FDA approved commercial products at present involve plasma fractionation devices [45].

### 5.1. Plasma Fractionation Derived Fibrinogen with Calcium and Commercial Thrombin

A wide variety of devices are now available to assist with obtaining platelet-poor or platelet-rich plasma which can be used as a source of nonconcentrated fibrinogen (20–40 mg/mL) and combined with one of the commercially available free standing thrombins (Thrombin-JMI, King, Pfizer, UPM Pharmaceuticals, Bristol, TN; Evithrom, Ethicon/J&J, Somerville, NJ; Recothrom, The Medicines Company, Parsippany, NJ) to obtain fibrin sealant. The available devices (including Amicus, Baxter, Round Lake, IL; Cell Saver, Haemonetics, Braintree, MA; Harvest, Smith and Nephew, Memphis, TN; Magellan, Medtronic, Minneapolis, MN; Recover, Biomet Biologics, Warsaw, IN; Symphony, Depuy, Raynham, MA) produce fibrinogen that when combined with calcium and thrombin produce fibrin sealant [45]. Calcium chloride is required to counteract the anticoagulant effects of sodium citrate often used to prevent clotting of the harvested plasma. Although this form of fibrin sealant uses a lower concentration fibrinogen than the commercially available concentrated pooled fibrin sealants resulting in less strength, it can produce a platelet enhanced fibrin sealant capable of possibly improving wound healing [86, 87].

## 6. Overview of Fibrin Sealant Literature Review

**Figure 2:**
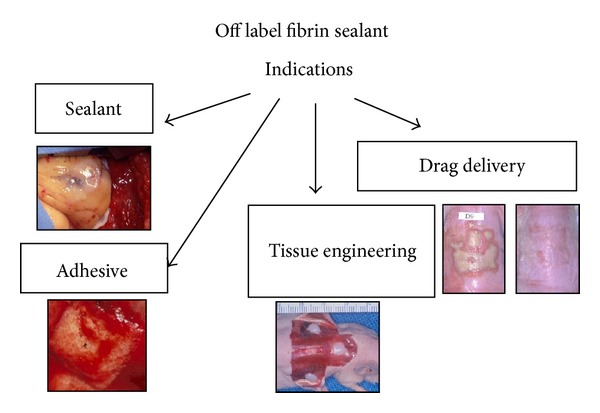
The off label indications for fibrin sealant [8], adapted and reprinted with kind permission from Springer Science + Business Media in [8, Page 633, Figure 2].

The fibrin sealant literature is quite extensive and continues to grow (approximately 200 papers/year). This literature in essence embodies not only the on-label applications (Figure 1), but also the off label uses of fibrin sealant as well (Figure 2). The goal here is to review the most recent past year's (February, 2012, through March, 2013) fibrin sealant literature in terms of the most frequent laboratory (Table 3) and clinical contributions (Table 4) as well as less frequent applications (Table 5). It is notable that all the off label indications (Figure 2) for fibrin sealant continue to grow in their number of references. While multiple papers have individually reviewed the fibrin sealant literature with reference to its use in specific specialties, only a few papers have reviewed this general overall literature in the past several years [8, 88–92] and none as recently or extensively as will be done in the following sections.

## 7. Fibrin Sealant Laboratory Literature

The latest laboratory work done on fibrin sealant consists of in vitro, ex vivo, and in vivo experiments and falls into two predominant descriptive bodies of research (Table 3): evaluations of material strength including sealing, adhesive, and internal bonding as well as other laboratory characteristics and tissue engineering with respect to mesenchymal or stromal cells and bone regeneration.

### 7.1. Sealing, Adhesive, and Internal Bonding Strength

These papers consist of studies designed to identify the best composition of commercial or experimental fibrin sealant to maximize sealant/adhesive strength. The presently available commercial formulations appear to have some role as sealants but may need additional modifications to be successful in all of the applications that investigators and clinicians envision.

A study comparing aprotinin containing fibrin sealant (FS-apr) and plasminogen reduced fibrin sealant (FS-rplg) noted significant in-vivo increases in median bursting strength in favor of FS-apr (50 mbar {range 30–55} versus 35 mbar {range 33–85}, *P* < 0.0001) in a rabbit, ileal intestinal, suture line model that included three lots of each sealant [93]. This difference was suggested by the authors to be possibly related to the fact that fibrin sealant containing aprotinin had thicker fibers on ultrastuctural evaluation more, *α*-chain cross linking on U-SDS-PAGE testing, and a longer lysis time on urokinase lysis.

Multiple different polypropylene meshes (Timesh Light, Ultrapro, and Optilene LP) employed for hernia repair were combined with different commercially available fibrin sealants (Tissucol, Quixil, and Evicel) to evaluate fixation strength. Researchers created an in vitro strength penetration test using a 45 mm defect in muscle tissue. It was determined by the test that each fibrin sealant had a particular best profile for which polypropylene mesh provided the strongest fixation: Tissucol—Optilene LP; Quixil—Optilene LP; and Evicel—Ultrapro. In addition, the three highest strength combinations of fibrin sealant and mesh were in descending order: Evicel—Ultrapro; Tissucol—Optilene LP; and Evicel—Optilene LP [94]. These authors suggested that the interaction between mesh and fibrin sealant was complex. It appeared as if the larger pore size mesh and the more slowly cross-linking fibrin sealant might have produced the strongest fixation.

A new test to evaluate the cohesive behavior of proteinaceous adhesives based on a “rigid double-cantilever beam” method was used to evaluate the strength of fibrin sealant and its components [95]. This method produces force-opening curves that can be used to derive detailed information on the strength of proteinaceous adhesives. Using this method, it was determined that both cross-linking by factor XIII as well as mixing with calcium improved the cohesive strength of fibrin sealant while not adversely affecting extensibility. This finding was true as long as calcium concentration was kept below 8 mM CaCl_2_.

An ex-vivo model using porcine lungs was employed to determine burst strength of uniform pleural defects to compare a variety of potential sealants [96]. Bioglue was found to have higher burst strength than Tisseel and Evicel fibrin sealants (*P* < 0.001) as well as TissuePatchDural (*P* < 0.05). Tachosil had higher burst strength than Tisseel (*P* < 0.001) and Evicel (*P* < 0.01) and Pleuraseal had higher burst strength than Evicel (*P* < 0.05). The authors determined that the liquid fibrin sealants used in this model had significantly less burst pressure than the Bioglue (albumin and glutaraldehyde), Tachosil (dry fibrin sealant patch), and Pleuraseal (polyethylene glycol polymer) (*P* < 0.05).

Using a canine model of liver resection, comparisons of fibrin sealant adhesion strength were performed [97] which determined that Tisseel (mean 775, range 491–1224 mN/m^2^) had higher adhesion strength than Quixil (mean 246, range 148–409 mN/m^2^) (*P* < 0.002) or Beriplast (mean 242, range 153–384 mN/m^2^) (*P* < 0.001) and that Tachosil (mean 596, range 373–954 mN/m^2^) had higher adhesion strength than Quixil (*P* < 0.014) or Beriplast (*P* < 0.009). The authors suggest that the differences in adhesion strength may be influenced by the amounts of fibrinogen, thrombin, factor XIII, and collagen present in each product.

23-gauge needle holes in expanded polytetrafluoroethylene (PTFE) grafts were employed as a model to compare a sealant made of gelatin (G) derived from porcine skin and cross-linked with glutaradehyde (GA) with fibrin sealant (FS) (Beriplast). This study [98] determined that the method of application, specifically rubbing the sealant into the graft site strongly influenced the pressure burst strength of sealing with rubbing G-GA 400 mmHg and FS 200 mmHg and without rubbing FS 140 mmHg and G-GA 25 mmHg. Similar results suggesting an advantage in sealing strength to rubbing application of fibrin sealant as opposed to dripping or spraying have been published by others [99–101].

A computational model of fibrin sealant adhesion for cartilage implant fixation in the knee joint has been created which strongly suggests that fibrin sealant alone may not be strong enough to withstand in-vivo loading. However, the combination of fibrin sealant and chondrocytes may be more effective at preventing shear damage at the cartilage interface [102]. The authors suggest that further increases in material strength and compliance are still needed for materials to be used in this setting.

One additional publication [103] evaluated a new method for polymerizing fibrinogen utilizing “hole b” which acts strongly on fibrinogen *β* chains resulting in the production of fibrin with an increased porosity, viscoelasticity, strength, and degradation resistance. This form of fibrin network may be more receptive to cellular infiltration and tissue regeneration avoiding the difficulties of reduced fibroblast ingrowth associated with the high fibrinogen concentration of commercially prepared fibrin sealants. This paper emphasizes the differences between fibrin monomer production and the assembly of the monomers into a final polymer. The latter appears to be particularly important for tissue regeneration and engineering.

### 7.2. Additional Laboratory Characteristics Studies

These reports vary from evaluating the characteristics of autologous or salmon derived fibrin sealant to factors influencing fibrin sealant biodegradation.

Investigators evaluated fresh frozen apheresis plasma as a source of allogeneic single donor fibrin sealant using the methods provided in the Cryoseal System (CS-1, Asahi Kasei Medical Co., Ltd., Chiyoda, Tokyo, Japan) which employs cryoprecipitation to derive fibrinogen as well as precipitation by CaCl_2_ and ethanol to obtain thrombin [104]. Evidence supporting the manufacturing consistency and ability to obtain useful levels of clotting factors, particularly fibrinogen and thrombin was presented. Consistency was evaluated using different temperatures (2 to 8°C versus 34 to 37°C) for the starting plasma with a significant difference in thrombin concentration levels being detected (27.5 ± 17.5 IU/mL versus 41.2 ± 13.2 IU/mL, respectively, with *P* = 0.041). Forty-one units of plasma were used (individual volume 281 ± 23 mL) and each yielded a volume of components (12.4 ± 3.3 mL) including concentrations of fibrinogen (19.9 ± 4.6 mg/mL) and thrombin (25.7 ± 11.1 IU/mL). The study also reported that quality control requirements (5–20 mL yield) using the system during routine use over a three-year period (2008–2010) revealed success rates varying from 90.8 to 97.7%.

The antibody profile of Atlantic farm raised salmon derived fibrin sealant proteins was determined in a mammalian model using rats [105]. IgG and IgM antibodies developed to both the fibrinogen and thrombin components of the fibrin sealant, but native rabbit cross reactivity after a second exposure to the salmon fibrin sealant proteins was weak and no inhibitory/neutralizing effect was found on the host rabbit coagulation activity.

A report [106] evaluating the importance of stability in the success of scaffolds for cartilage regeneration in anterior cruciate ligament (ACL) injury repair of ex-vivo porcine knees determined that fibrin sealant might be of value in augmenting stability particularly in ACL deficient knees (*P* = 0.01).

Investigators [107] determined that a new fluorescein (fluorescein isothiocyanate) method of porcine fibrinogen labeling coupled with size-exclusion high performance liquid chromatography-fluorescence detection could be as effective as radioisotope (^125^I) labeling at determining the level of fibrinogen in beagle blood following intraperitoneal injection of fibrin sealant (*r* ^2^ = 0.989).

It has been found that high levels of fibrinogen bound thrombin (≥820 IU/mL) in clots exposed to cultured cells not only decrease local proliferation of human keratinocytes but may also cause apoptosis [108].

An in-vitro study determined that fibrinolytic activity in human bile caused by tissue plasminogen activator, bile acids, and other bile proteins produced a lytic effect on human fibrin sealant [109]. This increased fibrinolysis may decrease the effectiveness of fibrin sealant used in this clinical setting.

Using an isolation chamber model of an arteriovenous loop, it was reported that levels of fibrinogen in fibrin sealant have a significant effect on the amount of matrix degradation and vascular in growth [110]. Lower levels of fibrinogen (20 mg/mL as compared to ≥80 mg/mL) appear to increase matrix degradation (*P* < 0.05) and increase the amount of vascularized connective tissue (*P* < 0.05) present.

### 7.3. Tissue Engineering: Mesenchymal or Stromal Cell Growth, Bone Regeneration, or Other Tissue Regeneration

Multiple papers have been published in this area over the last year suggesting that this field of fibrin sealant investigation remains highly active and productive.

Five papers address the ability of fibrin sealant to successfully deliver stem cells as well as suggesting potential modifications in the fibrin sealant composition to improve its efficacy as a growth medium for tissue engineering. The first report found that human cord blood-hematopoietic cell derived three-dimensional scaffolds formed with fibrin (fibrinogen 20 mg/mL and thrombin 20 IU/mL) were most effective in both an in-vitro testing and an in-vivo murine transplantation model as compared to scaffolds formed from polycaprolactone or porous poly(lactide-co-glycolide) in terms of cell growth, maintenance of phenotype, morphology, and engraftment [111]. A second paper evaluated the long-term (day 56) therapeutic protein expression of differentiated adipose cells in-vitro using three-dimensional fibrin sealant scaffolds and found that fibrinogen concentration was important determinant [112]. Lower concentration (4 mg/mL) as opposed to higher concentrations (8, 40 mg/mL) of fibrinogen resulted in more adipocyte differentiation (*P* < 0.05). This finding was supported by a second publication [113] that found that equine mesenchymal stem cell migration into fibrin hydrogels had a much greater magnitude in both autologous and commercial fibrin sealant with lower fibrinogen precipitate solution percentage (25% superior to 50, 75, and 100%). Another report found that human adipose stromal cells could be successfully delivered using a fibrin sealant spray (Tisseel, Westlake Village, CA) and that these cells reached confluence and differentiation after 3 weeks in an in-vitro model [114]. An extensive review of fibrin sealant as a delivery system for mesenchymal stromal cells including bone, tendon and cartilage, ligament, heart, nerve, and wound healing applications suggested that more clarification of the interaction of stem cells, growth factors, and fibrin matrix is still needed prior to human trials [115].

Several papers explored the use of fibrin sealant to facilitate bone growth or reduce inflammation. The first paper used in-vitro cell culture and in-vivo mice subcutaneous implantation experiments to evaluate bone tissue engineering potential created by human mesenchymal stem cells derived from skin, bone, and dental follicles when combined with demineralized bone matrix in fibrin sealant (Greenplasts kit, GreenCross, Yongin, Korea) scaffolds [116]. The investigators compared the amount of in-vitro differentiation and the degree of in-vivo osteogenesis as measured by osteoblast gene expression. The dental follicles were found to have the largest gene expression (n.s.) and significantly higher calcium content (*P* < 0.05) than the other mesenchymal cells. Another report [117] found that growth factors (vascular endothelial and fibroblast) combined with human adipose-derived stem cells and fibrin sealant (fibrinogen 75–115 mg/mL, thrombin 4 IU/mL, and aprotinin 3,000 KIU/mL) in starch based scaffolds appeared to be capable of promoting bony tissue vascularization with minimal inflammation. This experiment was performed in an in-vivo mouse model. The results were determined for neovascularization by expression of specific growth factor markers while the results for inflammation were evaluated on the basis of histology and the presence of inflammatory cytokines. A third report [118] used an in-vitro model of human and porcine damaged intervertebral disc (IVD) tissues. After combination with Interleukin-1*α* to provoke inflammation, the amounts of proinflammatory cytokines were significantly (*P* < 0.05) reduced in the human and porcine IVD nucleus pulposus tissue embedded in fibrin sealant (fibrinogen 72–100 mg/mL, thrombin 400–625 IU/mL, and aprotinin 3,000 KIU/mL) (Spinal Restoration, Austin, TX, USA) suggesting a possible benefit to fibrin sealant injections to treat IVD pathology. Another group of investigators found that fibrin sealant with injectable calcium phosphate cement and bone morphogenic protein induced alkaline phosphatase and achieved stiffness similar to that of normal vertebral bodies at 8 weeks following implantation in rabbit model [119]. Similarly, a report in rabbits found that recombinant human bone morphogenic protein glycolic acid microspheres with fibrin sealant were superior to fibrin sealant or no agent controls at stimulating osteogenic activity [120]. In dogs, the largest improvements in indices of osteoblast activity were noted in a group undergoing transsutural distraction osteogenesis that received recombinant human bone morphogenic protein poly(lactic-co-glycolic) acid and fibrin sealant 5 days after operation as well as, in addition, recombinant human osteoprotegerin with fibrin sealant three weeks later [121]. Lastly, an allogenic bone, fibrin sealant, and mesenchymal stem cell combination was found to be significantly (*P* < 0.05) better than controls without stem cells at both bone length and formation in a rabbit tibial model designed to assess vertical bone augmentation [122].

Four other papers discuss the use of fibrin sealant in tissue regeneration using models in specific tissues. These include studies of using a fibrin patch to successfully deliver progenitor cells derived from cardiac adipose tissue into a murine model of myocardial infarction [123]; a cultured autologous suspension of keratinocyte in fibrin sealant (Tissucol Kit, Baxter, Austria) to significantly (day 3, *P* = 0.04, and day 7, *P* = 0.01) accelerate healing as measured by the degree of epithelialization in oral wounds in rabbits [124]; sheep respiratory epithelial cells on in-vitro fibrin gel (fibrinogen 5 mg/mL, thrombin 40 IU/mL) versus on collagen-coated microporous membranes [125] and finding no significant difference in proliferation, function, and differentiation suggesting based on these findings that the fibrin sealant might prove to be a usable scaffold capable of being molded into complex geometries required for tissue engineering of respiratory structures; and human adipose-derived stem cells with polylactic-co-glycolic acid scaffolds combined with collagen or fibrin sealant for in-vitro testing of bladder wall like characteristics finding that fibrin created the most desirable leaking/porosity properties [126].

## 8. Most Frequent Fibrin Sealant Clinical Literature Applications

The new fibrin sealant clinical literature can be grouped into five (Table 4) predominant applications presented in decreasing order of frequency of published papers: ophthalmology, hernia repair, noncardiac thoracic, fistula repair, and seroma prevention as will be covered in the following sections.

### 8.1. Ophthalmology

The number of contributions to the ophthalmology literature on fibrin sealant has been accelerating over the past 10 years with 21 papers published over the most recent year. The largest number of references concentrated on using fibrin sealant to assist with intraocular lens fixation. Successful use of fibrin sealant to secure positioning of a posterior chamber intraocular lens is a known technique in ophthalmology with the new contributions suggesting: reduced complications in a series (*n* = 50) of cases [127] versus suture fixation (7/25, 28% versus 14/25, 56%, *P* = 0.045); successful use [128] in children with the caveat that use in ectopia lentis in Marfan's Syndrome may have a high incidence of associated retinal detachment [129, 130]; intrascleral haptic lens fixation (for a previously placed posterior chamber intraocular lens) with corneal transplantation (penetrating keratoplasty or Descemet-stripping automated endothelial keratoplasty) for aphakic or pseudophakic bullous keratopathy [131]; and fixation of subluxated or decentered previously placed posterior chamber intraocular lens using the assistance of haptics [132, 133].

An in-vitro study reported that fibrin sealant (Tisseel VH Fibrin Sealant, Baxter Healthcare (Asia) Pte Ltd., Singapore) treatment of human Descemet's membrane by creating a network of fibers [134] significantly increased the membrane's hysteresis (*P* < 0.001) and flexural rigidity (*P* < 0.001) producing improvement in mechanical properties in terms of stabilizing and stiffening as well as bending rigidity which may allow for easier manipulation and less “rolling up” during selective corneal transplantation. Clinically, fibrin sealant (Tisseel, Baxter, Westlake Village, CA) has been used to seal microperforations in Descemet's membrane occurring during deep anterior lamellar keratoplasty [135] enabling the avoidance of penetrating keratoplasty.

A variety of clinical applications of fibrin sealant in ophthalmology are focused on sealing of tissues and the avoidance of sutures. These include sealing scleral patch grafts or fistula at the time of drainage tube placement for glaucoma [136, 137], placement of conjunctival-limbal autografts for pterygium treatment in children [138, 139] or for limbal stem cell deficiency [140], and conjunctival closure during trabeculectomy [141] or repair of fat prolapse [142]. One of these studies [141] was a nonrandomized prospective trial finding significant reductions in operative time (37.3 ± 4.4 min versus 34.0 ± 4.2, *P* < 0.006) as well as assessed pain at week 1 (4.7 ± 1.4 versus 3.5 ± 1.3, *P* = 0.002) and week 2 (2.6 ± 0.9 versus 1.5 ± 0.6, *P* < 0.001) using fibrin sealant. Two dehiscences occurred in the sealant group (*n* = 28) and none occurred in the sutured group (*n* = 29).

Four additional reports described successfully using fibrin sealant in a variety of settings. The first was a prospective, randomized study showing that in amniotic membrane grafting for treatment of pterygium, fibrin sealant significantly reduced operative time (17 versus 28 min, *P* < 0.05) as well as postoperative pain and discomfort [143]. Another employed an inner and outer layer of amniotic membrane surrounding a fibrin sealant patch to treat a large corneal perforation [144] while preparing for a later keratoplasty. A third reported treatment of a central buttonhole defect of a LASIK flap with associated epithelial ingrowth using fibrin sealant to prevent recurrent epithelial ingrowth [145]. The last paper reported a series of patients eyes (*n* = 139) in whom conjunctival redundancy was removed using fibrin sealant in a “paste-pinch-cut” technique where placement of fibrin sealant is used to secure the remaining conjunctiva [146]. There was one dehiscence reportedly due to failure of sealant polymerization.

### 8.2. Hernia Repair

The clinical research on hernia repair using fibrin sealant is predominantly centered on open repair of inguinal hernia and ventral hernia repair and laparoscopic or endoscopic inguinal hernia repair either as a totally extraperitoneal (TEP) or transabdominal preperitoneal (TAPP) approach.

A multicenter, prospective, randomized trial [147] of fibrin sealant versus traditional Lichtenstein repair for open inguinal hernia repair revealed statistically significant benefit of using fibrin sealant (*n* = 50) compared to conventional suturing (*n* = 52) in terms of operative time (median 31, range 28–35 min versus median 35, range 30–42.5 min, *P* < 0.05), numbness at week 1 (RR = 0.43, *P* < 0.01) and month 1 (RR = 0.17, *P* < 0.05), analgesia usage (*P* < 0.05), and postoperative pain at week 1 (*P* < 0.05) and month 1 (*P* < 0.05). There was one dehiscence in the fibrin sealant treated group. Another multicenter, prospective, randomized clinical trial of fibrin sealant (*n* = 159) versus traditional suture (*n* = 160) of small to medium sized hernia repair was performed by different group of investigators [148] because of excellent results and low levels of follow-up pain (0% severe, 2.7% moderate) noted in a large (*n* > 600) single center retrospective nonrandomized series [149]. Findings in the multicenter trial [148] included significant reductions in complications in the overall fibrin sealant group at year 1 (8.1%, CI 4.2–13.6 versus 14.8%, CI 9.6–21.5, *P* = 0.0344). When the one-year complication data was broken down into active (5.9%, CI 2.2–12.5 versus 13.1%, CI 7.2–21.4, *P* = 0.0329) and retired (12.8%, CI 4.8–25.7 versus 18.5%, CI 9.3–31.4, n.s.) subpopulations, the findings favoring reduced complications in the retired fibrin sealant treated group lost their significance. Additionally, no significant differences were found between the fibrin sealant and sutured groups for pain, hernia recurrences, wound healing complications, duration of surgery, length of stay, return to work time, or quality of life evaluations. A nonrandomized series [150] comparing fibrin sealant (*n* = 62) and sutures (*n* = 54) for mesh fixation in inguinal hernia repair found significant benefit in the fibrin sealant group for days to complete wound healing (8.13 ± 7.88 versus 12.80 ± 8.59, *P* < 0.001) and pain score 12 months following surgery (*P* < 0.001). A final paper [151] enrolled 87 high risk elderly patients (median age 81, range 70–92) with either diabetes mellitus (*n* = 38, 44%), coagulation pathology (INR > 2) (*n* = 33, 38%), grade B or C child's cirrhosis (*n* = 16, 18%), or dicumarol-treated cardiovascular disease in a trial of open hernia repair using mesh fixation with fibrin sealant. No complications including infection occurred and the majority of patients (85%) were same day discharges. However, disagreements between some authors continue over published results of fibrin sealant trials for inguinal hernia repair with respect to reductions in operative time, pain, complications, and recurrences [152, 153].

Reduction of seromas and adhesions in ventral hernia repair has also been a topic of investigation by multiple groups of investigators. A prospective nonrandomized single center study in patients undergoing laparoscopic repair [154] compared percutaneous injection of the ventral hernia sac with fibrin sealant plus wrapping using a compression bandage (*n* = 25) with wrapping using a compression bandage alone (*n* = 25). There were significant benefits favoring the fibrin sealant group for the incidence of seroma at week 1 (64% versus 92%, *P* = 0.017) and at month 1 (28% versus 72%, *P* = 0.002); seroma volume at week 1 (*P* = 0.002) and month 1 (*P* = 0.001); and abdominal wall normalization as determined by patients input at week 1 (52% versus 24%, *P* = 0.041) and at month 1 (88% versus 64%, *P* = 0.047). An in-vivo study in Wistar rats [155] performed by placing pieces of mesh to the abdominal wall found that no adhesions occurred to coated or uncoated polypropylene mesh and that fixation strength was stronger when uncoated mesh was used with sutures than with fibrin sealant (*P* < 0.01). The methodology, however, included the placement of only one drop of fibrin sealant placed at the middle of the patches as opposed to the sutures that were placed at the 4 corners of the patches. Another in-vivo study in pigs using laparoscopic placement [156] of polypropylene and polytetrafluorethylene (PTFE) meshes found that PTFE produced less visceral and omental adhesions than polypropylene and that fibrin sealant coating of the polypropylene and PTFE meshes prevented any adhesion formation. An in-vitro study of mechanical glue strength (stamp penetration) of polypropylene mesh glued to muscle [157] found that the fibrin sealant (64.33 ± 8.99 N) provided less strength (*P* < 0.001) than butyl-cyanoacrylate (105.35 ± 22.59 N) or bovine serum albumin cross-linked with glutaraldehyde (131.69 ± 24.03 N), although all three glues were significantly better than no fixation at all (2.94 ± 0.89 N, *P* < 0.001).

In the setting of laparoscopic TAPP hernioplasty, a prospective randomize single center trial [158] found no significant benefit of using fibrin sealant (*n* = 44) over staples (*n* = 45) in terms of postoperative chronic pain and quality of life. Both groups had a low incidence of recurrence (one in each group). A similar study comparing self-gripping or fibrin sealant placed mesh found no significant difference in postsurgical pain at one and three months and that the operation was statistically significantly shorter by 4.5 minutes (*P* < 0.006) using the self-gripping mesh [159].

Several papers report results treating inguinal hernias using laparoscopic or endoscopic techniques using TEP approaches with fibrin sealant mesh fixation. Laparoscopic technique was employed in 100 consecutive patients in one report [160] with no early recurrences and significant improvement in postoperative hernia pain score (*P* < 0.0001) as measured by the visual analogue scale (VAS), mobility (*P* = 0.01), and quality of life (*P* < 0.001). A large review with meta-analysis of laparoscopic TEP found similar recurrence rates for staple and fibrin sealant mesh fixation and a decrease in postoperative chronic pain in the fibrin sealant group [161]. A single center series (*n* = 472) using endoscopic TEP repair of inguinal hernias employing fibrin sealant mesh fixation [162] reported only 6 recurrences (0.9%) with most all occurring in patients treated early in the series. In another series of patients (*n* = 79) indirect inguinal hernias were specifically treated with an Endoloop (Ethicon Endo-Surgery, Inc., Blue Ash, OH) closure of the hernia sac and fibrin sealant mesh fixation with results described as: no postoperative recurrences, 1 seroma, 2 minor complications (wound infection successfully treated with oral antibiotics and testicular pain subsiding with time), and no chronic groin pain [163].

A recent review of ventral and inguinal hernias [164] including 36 studies using fibrin sealant mesh fixation reported that the published clinical evidence supported the fibrin sealant method of mesh fixation. The review suggested that the use of fibrin sealant was associated with shorter operative time and hospital stay as well as a lower incidence of chronic pain without increasing hematomas or recurrence rates.

Finally an in-vivo randomized prospective porcine model [165] was used to evaluate laparoscopic hiatal hernia repair using fixation of the acellular dermal matrix with sutures (*n* = 10) or fibrin sealant (*n* = 10). Statistically significant benefit for the fibrin sealant group was found for a reduction in operative time (74.7 ± 25.5 versus 127.0 ± 34.2 min, *P* < 0.01). At the time of necropsy (30 days) all meshes were intact and there was no significant difference in mean peel force between the groups (0.18 ± 0.08 versus 0.21 ± 0.17 N/mm, *P* = 0.49).

### 8.3. Noncardiac Thoracic Surgery

The recently published papers concentrate on treatment using mesh with fibrin sealant of pneumo-, chylo-, or hydo-thorax as well as tracheobronchial injury.

A large sequential series over a total of 5 years [166] compared patients (*n* = 377) treated for intraoperative air leaks undergoing resection for primary or metastatic tumors with fibrin sealant alone (*n* = 204) or with fibrin sealant plus a polyglycolic acid (PGA) sheet (*n* = 173). The fibrin sealant application was always done sequentially with rubbing of fibrinogen followed by rubbing of thrombin in the first group and rubbing of fibrinogen, application of the patch, rubbing of thrombin into the patch, and then rubbing of fibrinogen again on top of the patch in the second group. Statistically significant improvement in duration of pleural drainage (2.7 ± 1.55 versus 4.2 ± 2.10 days, *P* < 0.01) as well as incidence of prolonged (>1 week) air leak (0 versus 14 {6.6%} patients, *P* < 0.01) was found using the second method. A second group of investigators [167] performed a similar study in 126 consecutive video assisted thoracic surgery patients (VATS) using three different application methods of fibrin sealant and PGA sheets. They found that rubbing fibrinogen into the defect area followed by application of a PGA sheet sprayed with fibrin sealant (as compared to two other methods) had the best success at preventing postoperative air leakage (*P* < 0.05). This finding was also supported by a laboratory model that demonstrated a significantly higher seal breaking pressure (*P* < 0.05) for this third method of application as well. Another human study of sealing video assisted thoracic surgery (VATS) air leaks [168] with fibrin sealant and PGA sheets (fibrinogen rubbed into defect area followed by thrombin to create a seal then application of PGA sheet soaked in fibrinogen followed by adding drops of thrombin on top of the sheet) examined chest tube drainage in lower risk patients (*n* = 206). This group found that: the vast majority of patients (91%) had no air leaks allowing their chest tube removal on the first postoperative day; removal of chest tubes one day after cessation of any observed air leak (one day of prophylactic drainage) resulted in a low level of chest tube reinsertion for recurrent air leak (2.9%); and the need for recurrent chest tube insertion was greater in patients undergoing segmentectomy than lobectomy (*P* = 0.04). A case report of treating bilateral pneumothorax in a premature infant documented the use of a low calcium (0.59%) concentration fibrin sealant via the chest tubes to eliminate the air leaks and avoid reported complications such as hypercalcemia and bradycardia [169]. A rare case of pneumothorax associated with chronic graft versus host disease (cGVHD) was successfully treated with fibrin sealant pleurodesis under fluoroscopic guidance when prior treatments with autologous blood via chest tubes had failed [170]. This technique is similar to the one that was employed at the University of Virginia in 2000 [34].

A case report in a neonate with esophageal atresia and trachea-esophageal fistula who developed a chylothorax following primary repair and needed a repeat thoracotomy for treatment added to the existing literature supporting the use of fibrin sealant in this indication [171].

Another report of successful treatment appeared for a case of hydrothorax secondary to peritoneal dialysis using VATS placement of mattress sutures as well as PGA felt and fibrin sealant application [172].

Two reports of successfully treating membranous tracheobronchial rupture also appeared. One employed a combination of small pieces of adipose tissue and fibrin sealant delivered by a catheter through a bronchoscope [173] and the other employed a thoracotomy for tracheal reconstruction using a bovine pericardial patch with the patch suture lines sealed with fibrin sealant without the need for autologous tissue reinforcement [174]. An third case report illustrated the successful treatment of a bronchopleural fistula in a 3-week-old baby following a right lower lobectomy for congenital cystic adenomatoid malformation using bronchoscopic placement of porcine dermal collagen and fibrin sealant [175]. A final case report of treating massive hemoptysis due to invasive pulmonary aspergillosis by local bronchoscopic application of fibrin sealant [176] was published.

With respect to possible complications of fibrin sealant use in thoracic surgery, a case report suggested that an eosinophilic postoperative pleural effusion (14% eosinophils) was caused by fibrin sealant used to seal a pulmonary bullectomy excision line as documented by a positive drug lymphocyte stimulation test [177]. However, the safety of fibrin sealant was supported by a multicenter, prospective, randomized trial of fibrin sealant [178] in patients (fibrin sealant *n* = 91, control *n* = 94) undergoing major thoracic surgical procedures that revealed a mortality of 1.1% in the fibrin sealant group and 5.3% in the control group. There were no tangible differences in the incidence of adverse events between the two groups (fibrin sealant 22% versus control 23.4%) including atrial fibrillation (fibrin sealant 5 versus control 4), hyperpyrexia (fibrin sealant 5 versus control 7), or thromboembolic events (none in either group). Thirty-seven percent of the fibrin sealant patients did develop antibodies to the bovine aprotinin contained in the fibrin sealant with no associated adverse events. There was a significant reduction in postoperative air leaks in the fibrin sealant group (9.52 versus 35.8 hrs, *P* < 0.005), but no significant reduction in the time to chest tube removal.

### 8.4. Seroma Prevention

This capability of fibrin sealant remains highly controversial. In my opinion the conflicting evidence relates to the concept of how to effectively employ fibrin sealant either as a sealant/adhesive to prevent serous drainage and seroma formation or as an antiadhesive that may actually increase serous fluid accumulation and seroma formation. The sealant/adhesive technique, although previously detailed in the literature [9, 13, 16, 17, 32, 36] has not been reliably employed in many studies. This lack of specific technique is critically important [32] in permitting the use of fibrin sealant as a sealant/adhesive that seals lymphatics as well as blood vessels and glues down flaps to underlying tissues. If this technique is not used the fibrin sealant works as an antiadhesive that prevents flaps from becoming adherent and maintains a potential space that facilitates increased serous drainage and seroma formation. The technique as described briefly again here requires these following measures. The fibrin sealant must be applied in such a manner as to assure wide surface distribution and allow for rapid and permanent skin flap placement. Mild pressure application on top of the flaps is required immediately after the application of rapidly polymerizing fibrin sealant spray (using thrombin 500–1,000 IU/mL) or as rapidly as possible after application of the more slowly polymerizing fibrin sealant spray (4 IU/mL). This is required because the tissues will only be glued together if they are in contact during the time of fibrin sealant polymerization (rapid polymerization 15–20 seconds, slow polymerization 60 seconds). Although the slowly polymerizing agent provides more time to align flaps before sealant polymerization, it runs the risk of fibrin sealant liquid pooling. This pooling may occur if slow polymerization permits gravity guided run off of the fibrin sealant liquid into the most dependent portions of the wound thereby decreasing overall wound sealing and adhesion. This risk is particularly important in a wound that has a variety of topographic configurations. In our hands all of this has meant that a lattice work of flap closure sutures must be preplaced prior to rapidly polymerizing fibrin sealant application. After applying the fibrin sealant the lattice work is quickly tightened and tied followed by pressure application to the flaps for at least 2 minutes. This method of wound closure is required as later wound closure interventions including manipulation of the skin flaps in any way after fibrin sealant polymerization results in permanent breaking of the fibrin sealant bonds. The resultant non-adherent fibrin sealant occupies the space between the underling tissue and flaps maintaining their separation. Fibrin sealant is capable of functioning as an adhesive (when in immediate contact with opposed tissues) [179] or (following a delay in tissue contact or a breaking of initial bonding) as a smooth nonreactive substance that has been shown to perform well as an antiadhesive [180] by the same group of investigators. This fibrin sealant antiadhesive keeps the underlying tissue and flaps separated for a prolonged period of time encouraging the formation of serous fluid drainage and seroma formation into the open potential space. If lymphatics and blood vessels are not sealed and the underlying tissue and skin flaps have not become adherent or if their adhering bonds are broken at any time, the fibrin sealant will fail to reduce serous drainage and seroma formation. In fact in this situation, both the serous drainage and seroma formation will be larger than if fibrin sealant had not been used at all. As will be described in the following paragraphs, it appears as though some investigations may continue to be performed without using these key concepts or the above-described technique apparently contributing to some lack of success in reduction of serous drainage or seroma prevention.

This past-year's literature on using fibrin sealant continues to reflect the controversy on its use to prevent serous drainage and seroma formation. At the latissimus dorsi harvest site during breast reconstruction following mastectomy, two papers support its use [181, 182] in conjunction with quilting sutures and two papers suggest that it does not work when used alone [183, 184]. In a single center nonrandomized sequential trial of using fibrin sealant alone (*n* = 25) versus quilting sutures with sprayed fibrin sealant (*n* = 21) conducted over a period of 5 months, significant reductions in the incidence of seroma (76% versus 42.9%, *P* = 0.022), seroma volume (median 30, range 20.0–46.7 versus median 45.0, range 25.0–160.0 mL, *P* = 0.043), total drainage (median 754.8, range 623.7–925.2 versus median 1,228.6, range 824.2–2,078.0 mL, *P* = 0.002), indwelling period of drainage (12.1 ± 3.9 versus 15.8 ± 5.9 mL, *P* = 0.01), and frequency of aspiration (4.8% versus 12%, *P* = 0.043) was found in favor of using quilting sutures and fibrin sealant [181]. The authors suggested that the quilting sutures stabilize the potential space between the skin flaps and the underlying tissues. It is possible that this stabilization by quilting sutures placed and tied before fibrin sealant application prevents shearing forces from breaking the established bonds of fibrin sealant between tissue planes preventing the sealant from becoming an antiadhesive. A second retrospective series described similar finding comparing a quilting alone group (*n* = 19) and a quilting with fibrin sealant group (*n* = 23) in favor of the quilting with fibrin sealant [182] in terms of indwelling period of drainage (mean 13.9, range 6–38 versus mean 21.5, range 9–26 days, *P* < 0.05), but not seroma rate (4% versus 5%, *P* = 0.4). The quilting sutures were carefully placed and tied to take tension off of the wound closure prior to using the fibrin sealant. Wound closure was then performed after fibrin sealant application using the drip applicator up under the previously placed quilting suture fixed flaps. This method appears to reduce the likelihood of disrupting the fibrin sealant bonds during manipulation of the wound edges. Several reports described the lack of fibrin sealant efficacy including a nonrandomized study of patients by a single surgeon over 5 and 1/2 years [183] and a single center, prospective, randomized trial conducted over 4 years [184]. In the first study no significant benefit to using fibrin sealant was found [183] as drainage in the control group (*n* = 23) was higher than in the fibrin sealant group (*n* = 23) until postoperative day 2 but beginning on postoperative day 3 and thereafter the control group had less drainage (*P* = 0.678) and the drain was removed earlier in the control group (11.8 versus 12.8 days, *P* = 0.339). In this study no description of the final skin closure and its possible movement of the fibrin sealant adherent skin flaps is made. It is possible that the fibrin sealant bonds were partially broken during skin closure producing an antiadhesive effect. In the second study [184] consisting of control (*n* = 53) and slow setting fibrin sealant (*n* = 52) treated groups powered for size effect, there were no significant differences with respect to the fibrin sealant and control groups, respectively, in seroma volumes (median 578, range 0–2,557 versus median 550, range 0–3,423, *P* = 0.348), total drainage volumes (median 1,700, range 566–4,562 versus median 2069, range 542–3836, *P* = 0.663), or seroma formation (4 versus 3 aspirations, *P* = 0.246). Interestingly grade I/II skin necrosis trended towards reduction in the fibrin sealant group (4% versus 7%, *P* = 0.118) following drain removal. Although the authors state that slow setting fibrin sealant was sprayed, the concentration of thrombin was reported as 500 IU/mL (slow setting commercially available fibrin sealant has a thrombin concentration of 5 IU/mL) making it unclear as to the type of fibrin sealant (fast or slow setting) actually employed. In addition, the authors state that two anchoring sutures were placed on either side of the skin closure about 2 cm away from the wound edge and were not tied until after the fibrin sealant was applied. This method again raises the issue as to whether the skin flaps were significantly manipulated after the fibrin sealant application during the tying of the two flap sutures up under the flaps causing a break in the fibrin sealant bonding of the flaps to the underlying tissues producing an antiadhesive fibrin sealant effect. These four papers appear to strongly reinforce the concepts previously discussed at the start of this section with respect to knowing the importance of how to use of fibrin sealant as a sealant/adhesive or as an antiadhesive.

Another report [185] described no statistically significant advantages for using fibrin sealant to reduce drainage following thyroidectomy, although trends in favor of fibrin sealant drainage reduction were found (at 24 hours, 64.3 ± 17.5 mL versus 73.0 ± 18.0 mL, *P* = 0.06; total amounts, 93.5 ± 30.7 mL versus 105.7 ± 31.2 mL, *P* = 0.05). The description of the operative procedure states that the fibrin sealant was sprayed, drains were then placed, and finally the wound was closed layer-by-layer suggesting a possible delay between sealant application and flap apposition as well as the potential for bonding disruption. Both techniques could have led to an antiadhesive effect of the fibrin sealant.

Two additional reports of successful use of fibrin sealant to reduce serous drainage appeared. The first was a prospective, randomized, single center study of radical lymph node dissection (axillary or ilio-inguinal) where a fibrin sealant coated equine collagen patch was placed over the entire wound prior to skin closure [186]. Significant benefits in the treated (*n* = 33) versus the nontreated group (*n* = 37) included reduction in drainage time (20.1 ± 5.1 versus 23.3 ± 5.1 days; *P* = 0.010) and percent of patients without drains at day 21 (86% versus 67%; *P* = 0.049). The authors point out that this patch was activated after 3–5 minutes of pressure. A case report of using lymphatic mapping, quilting sutures, and fibrin sealant to close a Morel-Lavallee lesion (persistent seroma formation following a degloving injury) of the thigh [187] described successful treatment with no recurrence at 6 months.

A prospective, randomized, single center report [188] performed over one year as well as a separate meta-analysis [189] found a lack of success using fibrin sealant to reduce serous drainage following mastectomy. The first [188] found trends, but no statistically significant benefit of using fibrin sealant (*n* = 31) as compared to control (*n* = 29) to reduce seroma formation (16.1% versus 24.1%, *P* = 0.43). The authors did note several findings in the fibrin sealant group: a baseline trend towards an increase in patient weight (74.6 ± 32.3 versus 71.7 ± 21.5 kg, *P* = 0.1273), a significantly reduced seroma aspirate volume (110 versus 210 mL, *P* = 0.0015), and a trend towards reduced day of seroma resolution (17 ± 8.9 versus 18 ± 8.6 days, *P* = 0.0968). The fibrin sealant spray used was diluted twice (thrombin 125 IU/mL) and sprayed just prior to skin closure of a loose lattice work of sutures that was then followed by even pressure to the wound for 5 minutes. This method may have allowed the more slowly polymerizing fibrin sealant to run off to the more dependent portions of the wound before polymerizing resulting in an uneven seal. The meta-analysis [189] suggested that thus far fibrin sealant has failed to prove a benefit in terms of postoperative seroma formation, average seroma volume, wound infection, complications, and length of stay. The authors recommended a new, controlled, large, multicenter, prospective, randomized, clinical trial to resolve this issue. At least one such trial already exists finding significant reductions in serous fluid drainage and days of drainage without increases in wound infection or complications in an FDA monitored clinical trial [36].

A rat study [190] found that triamcinolone acetonide (*n* = 12) was superior to fibrin sealant (*n* = 12) at reducing seroma volume (4.04 ± 1.43 mL versus 8.51 ± 2.60 mL, *P* < 0.05). A prospective, randomized, clinical study performed in a single center over a period of 7 months found the fibrin sealant spray group (*n* = 15) to have significantly higher seroma formation than the do nothing (*n* = 15) and quilting suture groups (*n* = 13) in abdominoplasty procedures [191]. No description of the timing of the fibrin sealant application, method of wound closure, or pressure application was provided.

### 8.5. Fistula Closure

Only one multicenter, prospective, randomized, clinical trial involving the use of fibrin sealant for fistula closure appeared in 2012. It [192] involved using autologous adipose-derived stem cells alone (group A) or fibrin sealant in combination with cells (Group B) or fibrin sealant alone (Group C) to treat fistula-in-ano in combination with suture closure of internal fistula opening (*n* = 165). In this phase III trial, there was no statistically significant difference in the healing rate between the three groups 39.1%, 43.3%, and 37.3%, respectively, at 24–26 weeks (*P* = 0.79). At one year the rates were 57.1%, 52.4%, and 37.3% (*P* = 0.13) suggesting a possible trend towards a fibrin sealant benefit. Selective analysis of the initiating center's data alone found significant benefit to treatment with cells and fibrin sealant at 24–26 weeks with healing rates of 54.55%, 83.33%, and 18.18% (*P* = 0.007). A multivariate analysis revealed that treatment center and fistula severity were independent predictors of fistula healing (*P* = 0.024). Also analysis of those patients receiving cells with or without fibrin sealant versus fibrin sealant alone revealed a doubling of the healing rate (*P* = 0.048) in favor of those patients receiving at least some cells. A trend towards antibiotic use as being significant was also noted (*P* = 0.09). There were no significant adverse events. However a retrospective follow-up study [193] from the initiating site itself of patients enrolled in an earlier phase II study unfortunately revealed a low proportion of patients without a recurrence after 3 or more years of observation. Two retrospective series of patients found benefit of using fibrin sealant to close anal fistulae [194, 195]. In the first study over a period of 5 years, occlusion of fistula tracts in patients with Crohn's disease (total *n* = 25) using fibrin sealant or cyanoacrylate (cyanoacrylate in only the first two patients as vulvar ulceration was noted following use in second patient) persisted in only 26% of treated patients for more than 6 months using application via an endoanal ultrasonographic-assisted percutaneous transperineal approach (initially 90.5% success at 4 weeks). In the second study over a period of 6 years [195], consecutive patients (*n* = 28) with transsphincteric cryptoglandular fistulas were treated in two stages. The first stage was a seton assisted fistulectomy of the main tract and the second stage involved insertion of fibrin sealant into remaining secondary segments of the tract. At a mean of 20.6 months, 67.8% had no recurrence (range 3–60 months) and none of these patients developed incontinence. A third large retrospective series [196] of patients over 34 years found a continuing trend towards more conservative non-cutting procedures and away from fistulotomy over time including using fibrin sealant, although results with fibrin sealant produced some lower long term success rates and have been used less in the last five year period. A meta-analysis found that the use of fibrin sealant as a fistula plug was less effective than conventional surgical management [197]. A final review paper of treatment for fistula-in-ano [198] continued to describe fibrin sealant as initially having good results, but it did not maintaining longer term success. It was suggested that it might still be useful to employ fibrin sealant as part of a conservative regimen of treatment including seton assisted fistulotomy.

Recent case reports of successful closure of intestinal tract fistulas using fibrin sealant include endoscopic instillation for treatment of esophageal perforation secondary to a fish bone [199], platelet-poor concentrated plasma closure of a percutaneous endoscopic gastrostomy fistula [200], platelet-rich concentrated plasma placement via a tract catheter for closure of a colocutaneous fistula [201], and endoscopic treatment of a gastro-jejunal fistula with fibrin sealant spray and a vicryl mesh plug [202].

## 9. Less Frequently Published Clinical Applications of Fibrin Sealant

Multiple additional papers address a variety of other topics and will be presented here in descending order of frequency (Table 5) for the use of fibrin sealant in the form of platelet-rich (PRP) or platelet-poor plasma (PPP) [203–213], hepatic surgery [214–222], neurosurgery [223–233], joint replacement orthopedic surgery [234–241], head and neck surgery [242–248], vascular surgery [61, 249–252], gastrointestinal surgery [253–258], gastroesophageal surgery [259–262], other orthopedic surgery [263–266], renal surgery [267–271], cardiac surgery [272–274], dental surgery [275–277], hemophilia [278–280], obstetrical and gynecological surgery [281–283], pancreatic surgery [284–286], transsphenoidal surgery [287–289], bariatric surgery [290, 291], drug delivery [292, 293], endoscopic polypectomy [294, 295], plastic surgery [83, 296], skull base reconstruction [297, 298], complications [299], trauma [300], and urology [301].

### 9.1. Platelet-Rich or Platelet-Poor Plasma

The platelet plasma literature has several articles discussing the need for consensus on naming the various potentially available products [210] and their production sites and quality control [211]. Several in-vivo studies evaluate PRP in a variety of models including flexor tendon healing in rabbits when combined with fibrin sealant finding significant (*P* < 0.02) increases in healing strength at 2 weeks [204], skin flap survival in rats with significant benefit in survival, PRP > PPP > thrombin (*P* < 0.001) [203], and tooth regeneration in a porcine model using PRP and platelet-rich plasma fibrin with as a scaffold for dental buds [209]. Several papers compare and contrast PRP and leukocyte and platelet-rich fibrin (L-PRF) [212] or PRP, cryoprecipitate, and thrombin with commercial fibrin sealants [207] finding influences of leukocytes on growth factor efficacy and similar clot strengths, respectively. Several clinical articles discuss using PRP in a variety of surgical specialties: cardiac [213], general [213], gynecologic [213], rhytidectomy [206], and vaginal prolapse [208]. Finally, a review of both PRP and platelet-rich fibrin (PRF) was published [205].

### 9.2. Hepatic Surgery

In hepatic surgery, fibrin sealant has been evaluated and found to be an effective hemostat particularly in large resections [216, 220, 221], but it has not been successfully demonstrated to be a method of reducing postoperative complications [216, 218, 221]. A recent review of fibrin sealant in hepatic surgery, also supports the findings [217] that fibrin sealant may be an effective hemostat but thus far does not seem to reduce complications. One multicenter, prospective, randomized study [214] suggested that a patch composed of polyethylene glycol, trilysine amine, and oxidized regenerated cellulose (*n* = 32) was superior to a fibrin sealant-equine collagen patch (*n* = 18) in terms of median time to hemostasis (1.0 versus 3.0 min, *P* < 0.001) during hepatic resection. A rabbit study [219] of temporary occlusion and a separate large series of patients [222] with permanent occlusion supported the use of fibrin sealant for hepatic embolectomy. Finally, a case report of using fibrin sealant to facilitate preimplantation repair of a lacerated donor liver to assure successful transplant appeared [215].

### 9.3. Neurosurgery

The neurosurgical literature continues to support the use of fibrin sealant for dural closure in specific instances including duraplasty after suboccipital decompression for Chiari 1 malformation in conjunction with autologous pericranium [223], repair of dural tears associated with ossification of the ligamentum flavum [224], and a radiographically guided instillation for a nerve root tear which caused intracranial hypotension [225]. Dural repairs with fibrin sealant happen frequently enough that an effort to establish its appearance on MRI has been described [226] and it was concluded that water based sealants could not be differentiated from one another. A recent, review guide devoted to the treatment of spontaneous intracranial hypotension recommended fibrin sealant as one method to close an identified leak [227]. A case report used fibrin sealant to help seal a dural CSF leak causing a pseudomeningocele following lumbar disc surgery with a subsequent complete resolution of a concomitant abducens nerve palsy and diplopia [228]. Several papers address the role of fibrin sealant in nerve repair including an end to side nerve repair in rats [229] and a facial nerve repair in dogs [230] failing to find a fibrin sealant benefit and a favorable report of using a fibrin conduit for mesenchymal stem cells particularly in the presence of cylcosporine to enhance regeneration following peripheral nerve injury in rats [231]. Laser soldering in pigs [232] was described as superior to fibrin sealant for dural reconstruction as having higher burst pressure strength (98.00 ± 21.41 versus 70.80 ± 15.09 mmHg, resp.; *P* < 0.05). Finally, an in-vitro report of using nimodipine polyglycolic acid microparticles suspended in fibrin sealant as a depot system to treat posthemorrhagic cerebral vasospasm was published [233].

### 9.4. Orthopedic Joint Repair

In orthopedic surgery with respect to joint replacement, the main issue is whether or not fibrin sealant is capable of reducing blood loss and cost of total knee replacement (TKA). Two papers describing prospective, randomized trials support the use of fibrin sealant to reduce bleeding [234, 235]. Significant reductions in drainage output [234, 235], hemoglobin loss [235], need for transfusions [234, 235], time of functional recovery [235], and length of hospital stay [235] were noted. Another prospective, randomized series noted a significant reduction in drainage volume using fibrin sealant, but no reduction in cost [236]. A large, retrospective review (*n* = 400) found a significant reduction in drain output using fibrin sealant, but no benefit on hemoglobin levels, transfusion rates, or cost [237]. A double blind, placebo-controlled, prospective, randomized study [238] found no benefit to using fibrin sealant in TKA with respect to drain output or measurements of recovery. A sequential nonrandomized trial found no reduction in blood loss, cost, or length of stay using fibrin sealant [239]. A new method of measuring adhesive strength in joint repair was described [240] and in-vitro as well as in-vivo testing of this method using bone fragments and a bone scaffold made of hyaluronic acid, fibrin sealant, and PRP to facilitate one-step osteochondral repair was reported [241].

### 9.5. Head and Neck Surgery

In head and neck surgery, two papers described use of fibrin sealant in conjunction with polyglycolic acid sheets as a method of oral mucosal repair following cancer resections in multiple individual patients [242, 243]. This same method was investigated in a rabbit glossectomy model and was found to be useful as a potential replacement for tie over sutures or skin grafting [244]. Two separate laboratory studies supported the use of fibrin sealant in vocal cord reconstruction either alone as a means of increasing collagen deposition [245] or in combination with chondrocytes to maintain vocal cord vibration characteristics [246]. In a prospective, randomized clinical study (*n* = 40), patients treated with fibrin sealant coating of the tonsillar bed following tonsillectomy were found to have less postoperative inflammatory response as measured by circulating leukocytes, neutrophils, and cytokines [247]. Finally, a commentary highlighted the potential role of fibrin sealant with a porcine gelatin pledget and fibroblast growth factor to successfully close tympanic membrane perforations [248].

### 9.6. Vascular Surgery

The literature on using fibrin sealant in vascular surgery suggests that it can be successfully used to augment two-suture fish mouth end-to-side microvascular anastomosis in rats with a significant reduction in operative time (*P* < 0.001) with no changes in early or late graft patency [249], but a review article on sutureless anastomoses did not find good evidence to support the use of fibrin sealant alone for vascular anastomosis [250]. A large multicenter, prospective, randomized trial comparing fibrin sealant to pressure for control of bleeding at PTFE graft needle holes found a significant benefit in patients at minute 4 (62.9% versus 31.4%, *P* < 0.0001) [61]. A case report of repairing a complex carotid artery blow-out in recurrent thyroid cancer with fibrin sealant and multiple tissue layers appeared [251] as did a swine study supporting the efficacy of salmon derived fibrin sealant compared to kaolin coated gauze [252] in an established femoral artery hemorrhage model.

### 9.7. Gastrointestinal Surgery

The published literature in gastrointestinal surgery included two reviews on using fibrin sealant as a means of adjunctive anastomotic closure with the first suggesting possible benefit in ileal and gastric/bariatric anastomoses [253] and the second suggesting that it found no convincing results in terms of any benefit of using fibrin sealant external coating of colonic anastomoses [254]. An animal study in rats agreed with a lack of benefit of fibrin sealant at colonic anastomoses with no increases in burst strength or collagen deposition noted [255]. Two reports did support the use of fibrin sealant to prevent or treat fistulas. The first was a prospective, historically controlled trial of using PGA sheets and fibrin sealant to prevent pancreatic fistula formation at the time of laparoscopic-assisted gastrectomy [256] which found a significant reduction in fistula formation in the treated group patients (treated 0/34 versus control 3/34, *P* = 0.049) as well as amylase drainage fluid levels (POD1: treated 660 versus control 1220 U/L, *P* = 0.030; POD2: 270 versus 830 U/L, *P* = 0.038; POD3: 160 versus 630 U/L, *P* = 0.041). An animal study in rats of duodenal fistula found that fibrin sealant with gentamicin did not increase burst strength but did seem to have a trend towards a decrease in inflammation, adhesions, and abscesses on microscopic examination [257]. Finally, a small series of consecutive patients (*n* = 5) undergoing abdominoperineal resection had fibrin sealant used to reinforce the perineal closure with no wound complications at 6 months [258].

### 9.8. Gastroesophageal Surgery

Several papers evaluated the use of fibrin sealant to seal the mucosa of the esophagus or at esophagogastric anastomoses. Reports of using fibrin sealant to repair an esophageal penetration at the time of endoscopic myotomy as confirmed on repeat endoscopy [259] and using sutures and fibrin sealant to close the mucosa after endoscopic cricopharyngeal myotomy as confirmed at repeat esophagram [260] were published. A laboratory study in pigs (*n* = 6) supported the use of fibrin sealant to close the mucosa in a model of transesophageal, endoscopic, esophagomyotomy (TEEM), although the authors suggested that the procedure was not yet perfected enough for use in humans [261]. A rat model [262] demonstrated that in control versus fibrin sealant coated equine collagen patch treated animals, respectively, there was an increase in burst strength at esophagogastric anastomoses on days 0 and 3 (55.1 ± 4.6 versus 102.4 ± 7.3 mmHg, *P* < 0.010 and 19.7 ± 3.3 versus 34.6 ± 4.9 mmHg, *P* < 0.050), although no significant differences were noted in burst strength at days 5 and 7 (60.9 ± 18.2 versus 53.4 ± 6.6 mmHg, *P* = 0.690; and 118.8 ± 20.2 versus 97.2 ± 8.3 mmHg, *P* = 0.374).

### 9.9. Additional Orthopedic Surgery

In some additional new orthopedic surgery literature, fibrin sealant was found to be effective in a case report of arthroscopic, juvenile, allograft, cartilage implantation in the talus to secure the graft [263] and useful in a series of patients (*n* = 51) undergoing reconstruction of bone defects following resection of benign tumor and tumor-like lesions when combined with a biphasic ceramic resulting in consolidation in 50/51 patients at 56 months of followup [264]. In an animal study, fibrin sealant was found to be effective in combination with tendon-derived stem cells to promote repair in a rat patellar tendon window defect as measured by significant increases compared to control in ultimate strength, Young' modulus, and collagen fiber presence [265]. On the other hand, fibrin sealant was not found to be effective in improving the fixation of a press-fitted, cell-free, and collagen plug in an ex-vivo porcine knee model when compared to control without fibrin sealant as measured by worn surface area (11.7% versus 12.9%, *P* = n.s.) [266].

### 9.10. Renal Surgery

With respect to renal surgery, two reports discuss the use of fibrin sealant in stone removal. The first one describes using fibrin sealant in 107 cases of tubeless percutaneous nephrolithotomy as a method of obtaining hemostasis in the access tract [267] with no patients requiring a blood transfusion and with only one pseudoaneurysm requiring selective embolization. The second describes using a laparoscopic coagulum pyelolithotomy employing fibrin sealant to create a colored (methylene blue added) coagulum capable of helping to extract even more stones than originally documented on X-ray studies [268]. Fibrin sealant has also been used in total or partial nephrectomy including a case report of stopping hematuria from the ureteric remnant endoscopically using fulguration as well as fibrin sealant [269], a series of patients (*n* = 45) in whom autologous fibrin sealant was applied to the resection bed during laparoscopic partial nephrectomy [270], and another sequential retrospective series of laparoscopic partial nephrectomies [271] where microporous polysaccharide spheres (MPH, *n* = 12) or fibrin sealant (historical control, *n* = 43) was applied to the resection bed finding that mean estimated blood loss was significantly less in the MPH group (25.6 versus 86.3 mL, *P* = 0.036).

### 9.11. Cardiac Surgery

In cardiac surgery, there were 3 reports of using fibrin sealant: the first was a case report [272] of using a bovine pericardial patch and fibrin sealant to achieve hemostasis in conjunction with an intra-aortic balloon pump for posterior left ventricular rupture following a mitral valve replacement and Cox Maze IV procedure; the second was a randomized prospective series [273] performed over 6 months of injection of fibrin sealant (*n* = 42) into the bone marrow of sternotomy patients versus bone wax placement (*n* = 40) with statistically significant advantages to the fibrin sealant method (drainage first 24 hours, 186.67 ± 49.53 versus 333.75 ± 60.49 mL, *P* < 0.001; less total chest drainage, 326.19 ± 67.24 versus 516 ± 88.46 mL, *P* < 0.001; less packed red blood cell administration, 3.6 ± 1.25 versus 7.4 ± 2.13 U, *P* < 0.001; less fresh frozen plasma administration, 5.52 ± 1.64 versus 8.95 ± 1.77 U, *P* < 0.001; shorter intubation time, 40.36 ± 8.62 versus 46.25 ± 10.46 hours, *P* = 0.007; and shorter hospital stay, 10.45 ± 1.17 versus 11.03 ± 1.37 days, *P* = 0.045); the third and final report suggested that imbricated proximal sutures and fibrin sealant spraying during Bentall aortic root replacement are associated with low morbidity and mortality [274].

### 9.12. Dental Surgery

The dental surgeons published 3 papers: the first was a trial of fibrin sealant versus sutures, respectively, for periodontal flap closure [275] performed over a period of 2 months with each patient (*n* = 10) serving as their own control that found significant benefits on the fibrin sealant side flap at gingival biopsy on day 8 (fibroblasts, 70.45 ± 7.22 versus 42.95 ± 4.34, *P* < 0.001; inflammatory cells, 20.91 ± 4.46 versus 32.58 ± 4.29, *P* < 0.001; and number of blood vessels, 5.74 ± 2.41 versus 11.89 ± 3.64, *P* = 0.005); the second again involved patients (*n* = 33) serving as their own controls to investigate using biphasic calcium phosphate and fibrin sealant as a bone substitute versus autologous bone in delayed dental implantation for bilateral maxillary sinus floor elevation [276] finding similar new bone formation and success rates in both sinuses at one year postoperatively; the final report in rats studied fibrin sealant and bone marrow stem cells for alveolar bone regeneration at the site of traumatic alveolar bone loss [277] finding significant increases in new bone formation on Micro-CT scanning at 6 weeks as compared to negative and blank controls (both, *P* < 0.05).

### 9.13. Hemophilia Surgery

The hemophilia literature remains concerned about achieving hemostasis during and following elective surgical procedures. A historical series of 76 patients undergoing circumcision over a 21-year period was reported and fibrin sealant application was employed as an integral part of the operation since 2000 allowing a reduction in factor concentrate administration from a previous high of 6-7 days down to only 3 days perioperatively [278]. A case report noted the successful extraction of a tooth using a combination of tranexamic acid oral mouthwash as an antifibrinolytic for 7 days preoperatively with intraoperative fibrin sealant and absorbable figure of eight sutures without raising any tissue flap to achieve hemostasis without the need for any factor replacement [279]. A large literature review recommended always having fibrin sealant available when performing operative procedures on patients with hemophilia [280].

### 9.14. Obstetrical and Gynecologic Surgery

In obstetrics and gynecology, a report of using fibrin sealant to facilitate full thickness skin graft attachment at the time of vaginal reconstruction for distal vaginal agenesis appeared [281]. In another historically controlled (*n* = 15) prospective series of patients (*n* = 15) over 2 years, fibrin sealant was used as an adjunct to hemostasis on uterine closure sutures during laparoscopic myomectomy [282]. Significant benefit to using fibrin sealant was noted for operative time (47.7 versus 62.1 min, *P* < 0.05), time to achieve complete hemostasis (195.5 versus 361.8 sec, *P* < 0.0001), estimated blood loss (111.3 versus 230 mL, *P* < 0.05), and mean decrease in hemoglobin (1.36 versus 2.04 g/dL, *P* < 0.05). Lastly, in an experimental endometriosis induction model using endometrial autografts [283], fibrin sealant attachment of the grafts was found to be superior to sutures in terms of scores for adhesions (0.8 ± 0.7 versus 2.4 ± 0.8, *P* < 0.05) and inflammatory reaction (1.2 ± 0.7 versus 2.2 ± 0.8, *P* < 0.05).

### 9.15. Pancreatic Surgery

With respect to pancreatic resection, 3 papers appeared finding no reduction in the incidence of postoperative fistula following pancreatic resection. A large multicenter, prospective, randomized trial performed over 2 years found that a fibrin sealant equine collagen patch applied in patients (*n* = 275) during open or laparoscopic distal pancreatectomy had no significant effect on reducing the rate of postoperative fistula formation, although the amount of amylase in the surgical drains was significantly reduced on the first postoperative day (3,282 ± 6,067 versus 5,703 ± 9,322, *P* = 0.025) [284]. Similarly, a prospective, randomized trial (*n* = 57) of using external, anastomotic, fibrin sealant liquid application in pancreaticoduodenectomy found no significant benefit in terms of drain lipase levels, gastric or biliary leaks, wound infection, Clavien score of 3 or more, and hospital stay [285]. Finally, a report of a large series of hepatectomy (*n* = 228) and pancreatectomy (*n* = 113) patients with and without fibrin sealant spray to the pancreas or pancreaticojejunostomy found no benefit in terms of reducing fistula occurrence [286].

### 9.16. Transsphenoidal Surgery

With respect to transsphenoidal surgical procedures, a series of patients (*n* = 9) was reported [287] whose CSF leaks after endoscopic nasal approach to the skull base were successfully closed using endoscopic guided fibrin sealant injections into the sphenoid sinus cavity in awake patients (4 required lumbar CSF diversion as well). A case report described the closure of a CSF fistula from the cribriform plate of the right nasal cavity using a mucoperichondrial free graft and fibrin sealant in a patient who had previously undergone an elective septoplasty [288]. Finally, a retrospective review of patients undergoing repair of CSF leaks following skull base surgery using endoscopic repair with a pedicled nasoseptal flap with (*n* = 42) or without (*n* = 42) fibrin sealant found no benefit of using fibrin sealant in reducing recurrent postoperative CSF leakage [289].

### 9.17. Bariatric Surgery

In bariatric surgery, a report suggesting that surgeon experience may improve the outcome of gastric bypass surgery also noted that experienced surgeons were more likely to use fibrin sealant at a linear stapled gastrojejunostomy [290]. Another group reported the use of endoscopic injection of fibrin sealant (EIFS) as a safe and successful means of treating leaks following Roux-en-Y gastric bypasses without the need for reoperation in any treated patients [291].

### 9.18. Drug Delivery

Two papers described the use of fibrin sealant as a means of drug delivery, specifically antibiotics in orthopedic implant models. The first used cefazolin, fusidic acid, or 5-fluorouracil with fibrin sealant and found in-vitro agar plate bacterial killing with each drug. In addition, significant reductions in infection as measured by clip bacterial loads and local swelling with each drug were found in an in-vivo Staph. aureus contaminated rat model of titanium spine clip implantation [292]. The second paper found similar results suggesting a benefit to combining vancomycin with fibrin sealant on in-vitro agar plates [293]. The investigators also performed a sequential clinical study comparing surgically placed orthopedic hardware without (*n* = 188, 2003–2007) and with (*n* = 196, 2007–2010) local application of the antibiotic impregnated fibrin sealant and found significant reductions in the rates of surgical site infection in the antibiotic fibrin sealant hardware group (0/196, 0% versus 11/188, 5.8%, *P* = 0.0003) [293].

### 9.19. Endoscopic Polypectomy

Two papers suggested using fibrin sealant during endoscopic polypectomy as a means of controlling hemorrhage. The first paper described methods of treating immediate bleeding that included hemoclips and fibrin sealant [294] and the second retrospective study (*n* = 344) suggested that fibrin sealant use based on multivariate analysis was associated with less delayed secondary hemorrhage following loop electrosurgical polyp excision (*P* < 0.01) [295].

### 9.20. Plastic Surgery

In cosmetic facial plastic surgery, a phase II, multicenter, prospective, randomized trial with the patients (*n* = 45) serving as their own controls was performed by using fibrin sealant to seal the skin to the underlying tissue on one side of the face and not the other [81]. Significant benefit of using the fibrin sealant was found in terms of reduced drainage at 24 hours (11.5 ± 13.7 versus 26.8 ± 24.0, *P* < 0.0001). Patient evaluation of pain, numbness, and preference also favored fibrin sealant. A review article of using fibrin sealant in rhytidectomy also appeared emphasizing the importance of proper technique in assuring that fibrin sealant functions as a sealant/hemostat promoting reduction of fluid production in this setting and not as an antiadhesive potentially allowing for increased fluid formation [296].

### 9.21. Skull Base Surgery

Skull base surgery research on fibrin sealant has involved studies evaluating burst strength measurement of the best ratios of fibrinogen and thrombin as well as evaluating the best grafts and sealants. The best in-vitro ratio was found using a high fibrinogen concentration fibrin sealant with a 5 : 1 ration of initial fibrinogen (i.e., 80 mg/mL × 5) and the usual thrombin concentration (250 IU/mL) to maximize adhesion strength [297] while the best in-vitro graft and sealant combination was found with fibrin sealant combined with pericranium (*P* < 0.0001) [298]. In the latter study, the difference between fibrin sealant and PEG polymer hydrogel with trilysine amine was not significant (*P* = 0.22).

### 9.22. Complications

A single report appeared of two different cases of small bowel obstruction following use of fibrin sealant at a laparoscopic Roux-en-Y gastric bypass which both required reoperation for resolution [299]. It was suggested that surgeons needed to carefully position bowel loops properly during and after using fibrin sealant to prevent bowel kinking.

### 9.23. Trauma

A comprehensive review article was published on the use of local and systemic hemostatic agents as an adjunct to control bleeding in trauma [300] that supported the use of fibrin based agents in some settings including coagulation disorders such as disseminated intravascular coagulation in which fibrinogen levels in the blood may be low and bringing exogenous fibrinogen to the bleeding site contained in the local hemostat may be especially useful.

### 9.24. Urology

Lastly, the use of fibrin sealant and purified porcine small intestinal submucosa mesh has been described in urology to reinforce the repair for transsphincteric posterior sagittal treatment of rectourinary and rectovaginal fistulas [301].

## 10. Conclusion

This spotlight paper has been designed to bring the reader up to date on the presently available forms of fibrin sealant as hemostats, sealants, and adhesives as well as to provide an organizational structure for contrasting these forms of fibrin sealants with other available products. In addition, a review of a year's publications on fibrin sealant has been provided based on both laboratory and clinical studies. These reports have been subdivided into areas that have been presented based on frequency and reported in order from most frequent to least frequent occurrence.

## Figures and Tables

**Table 1:** A system of classification for FDA approved local hemostats, sealants, and adhesives in 2013. The fibrin sealants are in bold font [45], adapted and reprinted with kind permission of the Southeastern Surgical Congress in [45, page 1306, Table 1].

Group	Category	Class
Hemostats	Mechanical	Porcine gelatin
		Bovine collagen
		Oxidized regenerated cellulose
		Polysaccharide spheres
	Active	Bovine thrombin
		Human pooled plasma thrombin with or without porcine gelatin sponge or powder
		Recombinant human thrombin
	Flowable	Bovine gelatin and human pooled plasma thrombin
		Porcine gelatin ± thrombin
	**Fibrin sealant**	**Human pooled plasma *liquid***
		**Individual human plasma *liquid*, bovine collagen, and bovine thrombin**
		**Human pooled plasma and equine collagen *patch***
		**Human pooled plasma and oxidized regenerated cellulose *patch***

Sealants	**Fibrin sealant**	**Human pooled plasma liquid**
	Polyethylene glycol polymer (PEG)	Two PEGs
		PEG, trilysine amine, and FD&C Blue #1
		PEG and human serum albumin
	Albumin & glutaraldehyde	Bovine serum albumin and 10% glutaraldehyde
	Cyanoacrylate	Octyl and butyl lactoyl cyanoacrylate

Adhesives	Cyanoacrylate	Octyl cyanoacrylate with FD&C Violet #2
		Octyl cyanoacrylate with FD&C Violet #2 and polyester mesh
		Butyl cyanoacrylate with or without FD&C Violet #2
	Albumin & glutaraldehyde	Bovine serum albumin and 10% glutaraldehyde
	**Fibrin sealant**	**Human pooled plasma *liquid***

**Table 2:** The available forms of fibrin sealant in the USA in 2013.

Function	Source	Product
Hemostat and sealant	Human pooled plasma	*Tisseel*, Baxter, Westlake Village, CA
Hemostat	Human pooled plasma	*Evicel*, Ethicon/J&J, Somerville, NJ
Hemostat	Individual plasma, bovine collagen and thrombin	*Vitagel*, Orthovita/Stryker, Malvern, PA
Hemostat	Human pooled plasma and equine collagen	*Tachosil*, Baxter, Westlake Village, CA
Hemostat	Human pooled plasma and oxidized regenerated cellulose	*Evarrest*, Ethicon/J&J, Somerville, NJ
Adhesive	Human pooled plasma	*Artiss*, Baxter, Westlake Village, CA

**Table 3:** The most frequently published topics of fibrin sealant laboratory research.

Most frequent topics of fibrin sealant laboratory research
Characteristics
Strength (sealing, adhesive, internal bonding)
Tissue engineering
(1) Mesenchymal-stroma cells
(2) Bone formation

**Table 4:** The most frequently published topics of fibrin sealant clinical research.

Most frequent topics of fibrin sealant clinical research
Ophthalmology
Hernia repair
Noncardiac thoracic
Seroma prevention
Fistula repair

**Table 5:** Additional topics of fibrin sealant research presented in decreasing order of frequency of publication.

Additional topics of fibrin sealant research (descending order of frequency)		
(1) Platelet plasma (2) Hepatic surgery (3) Neurosurgery (4) Joint replacement surgery (5) Head and neck surgery (6) Vascular surgery (7) GI surgery (8) Gastroesophageal surgery (9) Orthopedic surgery	(10) Renal surgery (11) Cardiac surgery (12) Dental surgery (13) Hemophilia (14) Ob-Gyn surgery (15) Pancreatic surgery (16) Transsphenoidal surgery (17) Bariatric surgery (18) Drug delivery	(19) Endoscopic polypectomy (20) Plastic surgery (21) Skull base reconstruction surgery (22) Complications (23) Trauma (24) Urology
